# Metabolomics analysis for hydroxy-L-proline-induced calcium oxalate nephrolithiasis in rats based on ultra-high performance liquid chromatography quadrupole time-of-flight mass spectrometry

**DOI:** 10.1038/srep30142

**Published:** 2016-07-22

**Authors:** Songyan Gao, Rui Yang, Zhongjiang Peng, Hongtao Lu, Na Li, Jiarong Ding, Xingang Cui, Wei Chen, Xin Dong

**Affiliations:** 1 School of Pharmacy, Second Military Medical University, Shanghai 200433, China; 2 Brigade of undergraduate student, Second Military Medical University, Shanghai 200433, China; 3 Department of Nephrology, Changhai Hospital, Shanghai 200433, China; 4 Department of Urology, The Third Affiliated hospital, Second Military Medical University, Shanghai 200433, China

## Abstract

About 80% of kidney stones are composed of calcium oxalate (CaOx) with variable amounts of calcium phosphate, and hyperoxaluria is considered as an important factor of CaOx nephrolithiasis. However, the underlying metabolic mechanisms of CaOx nephrolithiasis remain undefined. In this study, we successfully developed a rat model with hydroxy-L-proline (HLP) -induced CaOx nephrolithiasis. Rats were continuously orally administrated with HLP for 28 days. Urine and blood samples were collected from the rats treated with or without HLP at four different time points. UPLC–Q-TOF/MS was applied to profile the abundances of metabolites. To obtain more comprehensive analysis of metabolic profiling spectrum, combination of RP-LC and HILIC were applied. We identify 42 significant differential metabolites in the urine, and 13 significant differential metabolites in the blood. Pathway analysis revealed that the pathways involved in amino acid metabolism, taurine metabolism, bile acid synthesis, energy metabolism, TCA cycle, purine metabolism, vitamin metabolism, nicotinic acid and nicotinamide metabolism have been modulated by HLP treatment. This study suggested that a number of metabolic pathways are dysfunctional in the HLP induced crystal kidney injury, and further studies on those pathways are warranted to better understand the metabolic mechanism of CaOx nephrolithiasis.

Nephrolithiasis is a common urinary tract disease which is often accompanied by symptoms of renal colic and hematuria in clinic. Nephrolithiasis is a risk factor of chronic kidney disease or end-stage renal disease, seriously affecting kidney function and even progressing to uremia^1^,^2^. Although nephrolithiasis is not a common cause of renal failure, certain problems, such as preexisting azotemia and solitary functional kidneys, clearly present a higher risk of additional renal damage caused by nephrolithiasis.

Stone formation of nephrolithiasis is a complex and gradual process, generally considered including crystal nucleation, growth, adhesion and aggregation of crystals^3^,^4^. However, the specific mechanisms of nephrolithiasis development have not been characterized. A low fluid intake, with a subsequent low volume of urine production, produces high concentrations of stone-forming solutes in the urine, which is an important environmental factor in kidney stone formation. Epidemiological studies have shown that the body’s metabolism dysfunction plays an important role in the formation of nephrolithiasis^5^,^6^. However the systemic metabolomics studies on nephrolithiasis are currently limited.

Overall, 80% of patients with nephrolithiasis form calcium stones, most of which are composed primarily of calcium oxalate or, less often, calcium phosphate^7^. Most research on the etiology and prevention of urinary tract stone disease has been directed toward the role of elevated urinary levels of calcium, oxalate, and uric acid in stone formation. An elevated level of urinary oxalate is considered as an important factor of CaOx nephrolithiasis. Oxalate metabolism in human and rats are considered basically the same^8^, and therefore rat model building stones can be used to study mechanisms of human kidney stone formation. A variety of animal models have been developed to investigate nephrolithiasis. Hyperoxaluria in rats induced by ethylene glycol (EG) or EG plus various concentrations of ammonium chloride or vitamin D is most common^9^,^10^, but is criticized because EG causes metabolic acidosis and some of its metabolites are also nephrotoxic and injurious to renal epithelial cells^11^,^12^. Thus, it is difficult to distinguish the effects of EG and its metabolites from those induced by CaOx crystals. Hydroxy-L-proline (HLP), a glyoxylate precursor^13^, is to produce glyoxylate in the mitochondria of liver cells or kidney proximal tubule epithelial cells^14^. The HLP, as an important component of collagen, often appears in our daily diet. Khan *et al.*^15^ have successfully developed an HLP-induced rat model of kidney stones, which is closer to human kidney stone formation process.

Recently, liquid chromatography - high resolution mass spectrometry (LC-HRMS) platform gradually had become the major analytical platform for metabolomics analysis, because of its high sensitivity, high throughput, tremendous versatility, as well as relatively simple sample preparation. The compounds with different polarity and molecular size can be analyzed by selecting the appropriate chromatographic column and adjusting the composition of the mobile phase. Reversed-phase (RP) LC with C18 columns are widely used, and provide reasonably good separation of nonpolar and weakly polar compounds. However, the major limitation is the lack of adequate separation of highly hydrophilic, ionic and polar molecules on an RP stationary phase. The hydrophilic interaction liquid chromatography (HILIC) has recently grown in popularity due to the high compatibility with MS and the need to improve detection of polar compounds. Urine contains lots of problematic polar molecules. Therefore, combined application of HILIC and RP separations is best-suited to cover a very large part of the urine metabolome, thus enhance the potential to generate more comprehensive urine metabolite profile than RP separation alone.

In the present study, we constructed a HLP-induced CaOx nephrolithiasis model with Sprague-Dawley (SD) rat and performed metabolomics analyses for the urine and serum samples at different time points after HLP treatment using UPLC-Q-TOF/MS. To obtain a more comprehensive metabolic profile, the data were acquired by using the combination of RP chromatography and hydrophilic chromatography. The differential metabolites were identified by the pattern recognition using OPLS-DA and univariate analysis for the control and HLP treated rats at the different time points. This study provided important data for the metabolic mechanism of HLP-induced crystal renal injury.

## Results

### Body weight, urine volume and urinary biochemical changes

**Figure 1:**
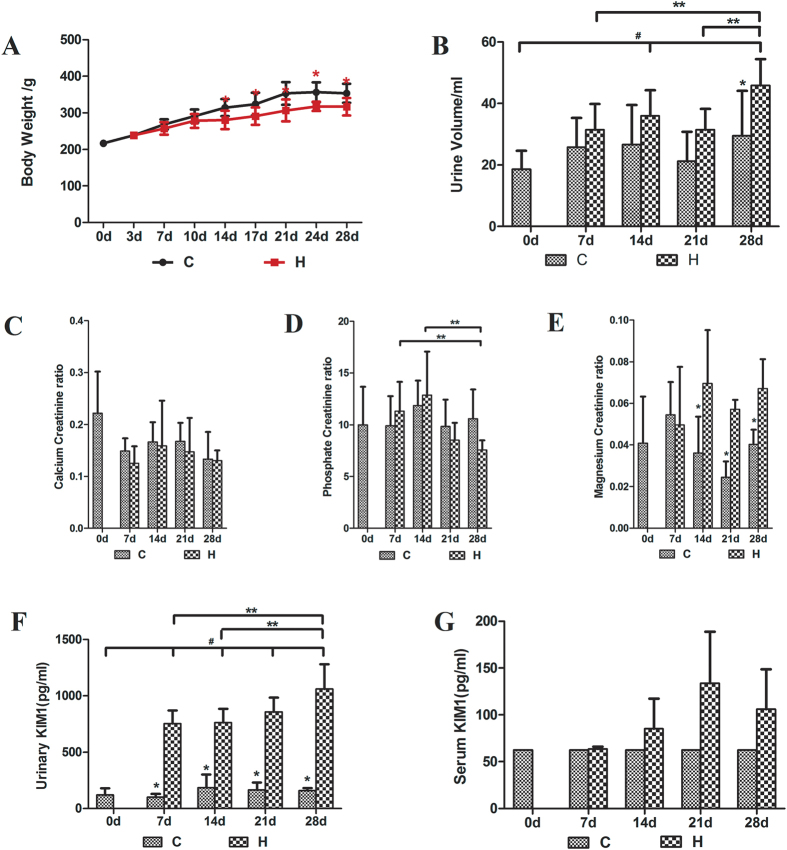
Effects of HLP on various physiological, biochemical parameters and KIM-1 levels in rats. (**A**) body weight ; (**B**) urine volume; (**C**) Urinary calcium creatinine ratio; (**D**) Urinary phosphate creatinine ratio; (**E**) Urinary magnesium creatinine ratio; (**F**) Urinary excretion of KIM-1; (**G**) Serum KIM-1 level (The amount of the control group is lower than the detection limit of the reagent box, and the values are expressed with the detection limit of the reagent box). *P < 0.05, between control and HLP, ^#^P < 0.05 between baseline and HLP, **P < 0.05 HLP between days.

One rat died at the day 28 of the experiment due to the unappropriated gavage, all other rats stayed healthy and gained weight. However, starting from the day 14, the HLP-treated rats (H group) gained statistically significantly less weight (*P* < 0.05), compared to the untreated control rats (C group) (Fig. 1A). In addition, HLP-treated rats produced significantly more urine than the controls (*P* < 0.05) (Fig. 1B), which is consistent with the previous reports^15^. No significant changes on calcium creatinine ratio were observed in HLP-treated rats and control rats (*P* > 0.05) (Fig. 1C). The urinary phosphorus creatinine ratio had significantly changed across different time points in H group (*P* < 0.05) (Fig. 1D), while urinary magnesium creatinine ratios significantly increased in HLP-treated rats, compared to those in the control rats (*P* < 0.05) (Fig. 1E).

### Urinary and serum KIM-1 levels

We determined the KIM-1 levels in urine and serum by an enzyme-linked immunosorbent assay (ELISA). Urinary KIM-1 levels in HLP-treated rats were significantly higher than that those in the control rats with a time-dependent manner (*P* < 0.05) (Fig. 1F). Significant differences on serum KIM-1 levels were observed between HLP-treated rats and control rats at the day 14, 21 and 28, respectively (*P* < 0.05) (Fig. 1G). However, no significant differences were found among HLP-treated rats at all studied time points (P > 0.05) (Fig. 1G). KIM-1 is an important indicator of renal epithelial cell injury. Significant increase of urinary KIM-1 and the accumulation serum KIM-1 indicated that the HLP treatment had a significant damage to kidney epithelial cells.

### Renal calcium deposition

**Figure 2:**
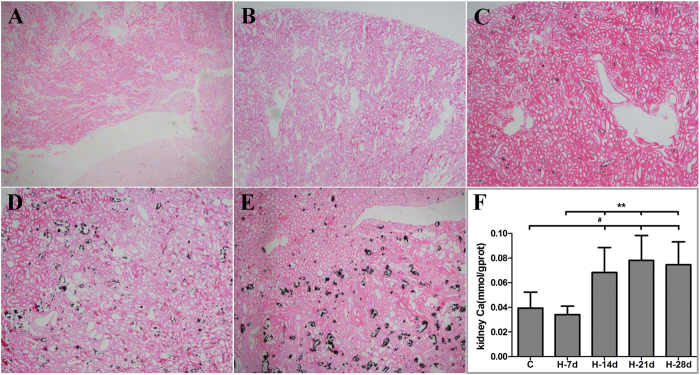
The Von Kossa Staining pictures and kidney calcium content of the Control group and HLP treatment groups. (**A**) control; (**B**) H-7d; (**C**) H-14d; (**D**) H-21d; (**E**) H-28d; (**F**) the kidney calcium content of control and HLP groups. ^#^P < 0.05, between control and HLP; **P < 0.05, HLP between days.

At the fourth week (day 28), the model renal tissue surface showing visible white spots, and kidney volume significantly increased. Von Kossa staining of kidney tissue showed that the renal tissue calcium deposits gradually increased with the growth of modeling time (Fig. 2A–E, black dots representing calcium deposits). Renal calcium content (Fig. 2F) under HLP further demonstrated the role from the beginning of the second week, a significant deposition of calcium renal tissue.

### Metabolic profiling analysis

RPLC-MS and HILIC-MS data of the urinary samples and RPLC-MS data of the serum samples were acquired based on the above methods. The representative total ion chromatograms (TICs) in positive mode of urine and serum from normal rats and HLP-treated rats were shown in Figure S1. Calculation urine sequence QC sample (n = 7) of each feature RSD (%) value, the variation of more than 80% features were less than 20%, indicating the stability of the system was satisfying. There were 544 and 673 features with RSD values less than 20% identified in RP-MS positive and negative mode, respectively. While 297 and 194 features with RSD values less than 20% were identified in HILIC-MS positive and negative mode, respectively.

**Figure 3:**
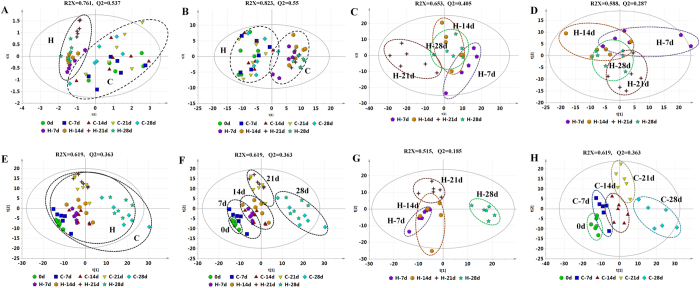
PCA score plots of urine (**A**–**D**) and serum (**E**–**H**) samples in the controls and HLP groups across different time points by RPLC-MS and HILIC-MS methods in positive mode. (**A**) Urine samples of all C groups and H groups based on RP-MS; (**B**) Urine samples of all C groups and H groups based on HILIC-MS; (**C**) Urine samples of H groups in four time points based on RP-MS; (**D**) Urine samples of H groups in four time points based on HILIC-MS; (**E**,**F**) Serum samples of all C groups and H groups; (**G**) Serum samples of all H groups; (**H**) Serum samples of all C groups.

Initially, unsupervised PCA was used to observe the separating trends and metabolic trajectories between each set of samples and eliminate outliers. One outlier in H-7d group from the RPLC-MS data set was observed and excluded based on the PCA score plots and the Hotelling’s T2 test (*P* < 0.05). Score plots from the PCA model have shown that the different control groups clustered together and were clearly separated from the HLP-treated groups (Fig. 3A,B). While the different HLP-treated groups exhibited an obvious separation trend and showed certain time-relied metabolic trajectories (Fig. 3C,D). In order to fully differentiate the metabolites between the control and the HLP-treated groups, the OPLS –DA model was applied. OPLS-DA is an efficient method to identify the ions that contribute to the clustering of samples. It also helps to eliminate non-correlated variations within the data set. There was a distinct clustering between the control group and the HLP-treated group in different time points both by RPLC-MS method (Figure S2) and HILIC-MS method (Figure S3). The *P* values from cross-validation analysis of variance (CV-ANOVA) were showed in Figure S4, indicating OPLS-DA models were valid. The corresponding S-plot showed the contribution of different variables for the differentiation between the control and the HLP-treated groups. Each point in the S-plot represents an ion. Ions far away from the origin are significant important to the differences between groups and have greater VIP values. Independent sample *t*-test was performed to assess the statistical significance. The important ions differentiating the HLP-treated group from the control group in different time points (VIP > 1, Fold Change >2 or < 0.5, p < 0.05) were identified according our previous methods^16^. Finally, 42 differentiated metabolites were identified in urine and listed in Table 1 and Table S1.

After pretreatment, a total of 619, 738 features in serum were obtained from the positive mode and negative mode, respectively. Furthermore, we applied the PCA model to characterize the differences between each group. The H groups didn’t show a good separation with the C groups (Fig. 3E), and the H group and the C group in each time point were clustered together (Fig. 3F). However, the H groups in different time point as well as the C groups in different time point were well separated (Fig. 3G,H). The results indicated that age deeply affected the serum metabolic profiling, even greater than the effect of HLP. Therefore, screening of metabolites with significant changes in HLP intervention must be ruled out by age.

We used a single variable Wilcoxon Mann-Whitney Test method to screen the significantly changed metabolites between the H group and C group in the same time points. Using the *P* < 0.05 and FC values greater than 2 as cut-off, 29 and 38 ions were observed in positive mode and negative mode, and 13 significant metabolites were identified eventually (see Table 2 and Table S2).

### The dynamic metabolic changes under the intervention of HLP

**Figure 4:**
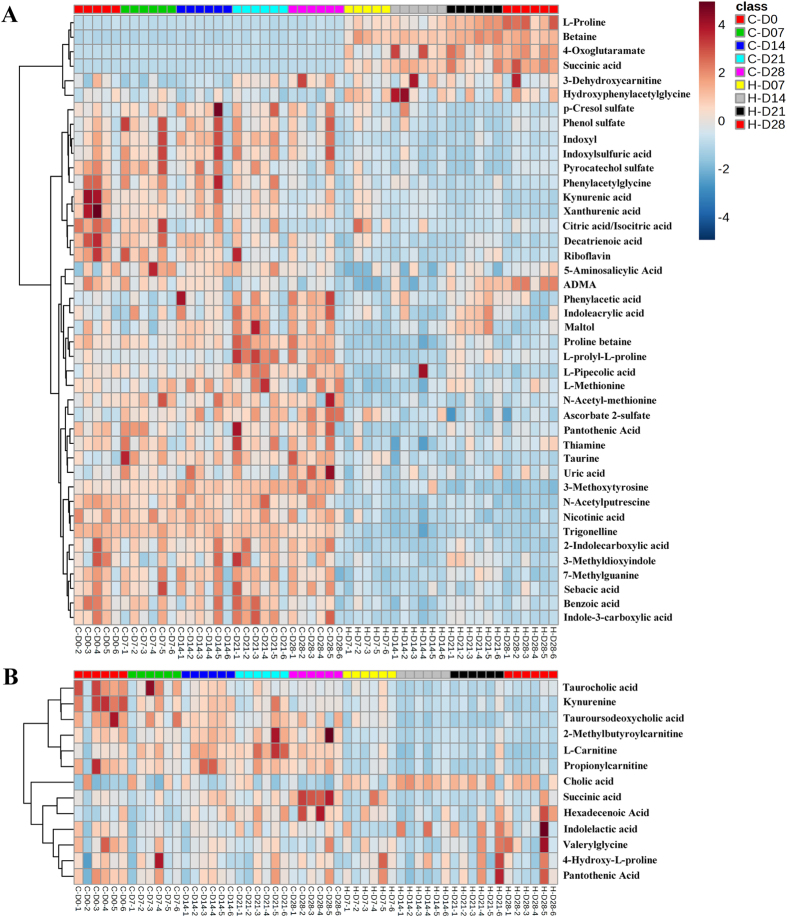
The clustering heat map of the control and HLP rats across different time points based on the 42 and 13 top differentially metabolites in urine (**A**) and serum (**B**), respectively. Each column is labeled with different colors according to the sample type.

Heatmap could visually display the dynamic changes of the differential metabolites between control groups and HLP-treated groups in different time points. Using the MetaboAnalyst platform (http://www.metaboanalyst.ca), the heatmps were constructed based on the normalized data set of the 42 differential metabolites in urine and 13 metabolites in serum (Fig. 4A,B). In urine, L-Proline, Betaine, 2-Oxoglutaramate significantly increased in HLP-treated groups, compared to the control groups. TCA (Succinic acid, Citric acid), Hydroxyphenylacetylglycine and 3-Dehydroxycarnitine were upregulated in each time point, and ADMA also significantly increased after the 21 days. The other metabolites, such as L-prolyl-L-proline, N-Acetylputrescine, proline betaine, taurine, uric acid, L-pipecolic acid, L-Methionine, N-Acetyl-L-methionine, 3-Methoxytyrosine, 7-Methylguanine, unsaturated fatty acids (Decatrienoic acid, Sebacic acid) were down-regulated. In addition, the metabolites involved phenylalanine metabolism (phenylacetic acid, phenylacetylglycine, benzoic acid), nicotinate and nicotinamide metabolism (nicotinic acid and trigonelline), and VB metabolism (pantothenic acid, thiamine and riboflavin), as well as tryptophan metabolism (indoxyl, 3-hydroxyanthranilic acid, 4,6-dihydroxyquinoline, 4,8-dihydroxyquinoline, 3- methyldioxyindole, indoleacrylic acid, kynurenic acid, indoxylsulfuric acid, xanthurenic acid) were down-regulated.

The serum metabolic profiling did not significantly change under HLP intervention, however, there still have significant changes in several metabolites. For example, Hydroxy-L-proline (HLP) was significantly up-regulated in each H group. Cholic acid, indolelactic acid, valerylglycine, pantothenic acid were also significantly elevated in the serum at multiple time points; In contrast with those in the urine, serum succinic acid significantly reduced; L-carnitine and propionylcarnitine involved in fatty acid transport and beta oxidation were significantly down-regulated after 14 days. Two conjugated bile acids, taurocholic acid and taurodeoxycholic acid, were significantly decreased in H-14d group and H-21d group, however no significant changes were observed in H-28d. Kynurenine, an important tryptophan metabolism related metabolite, also showed a down regulation in H-28d group.

## Discussion

**Figure 5:**
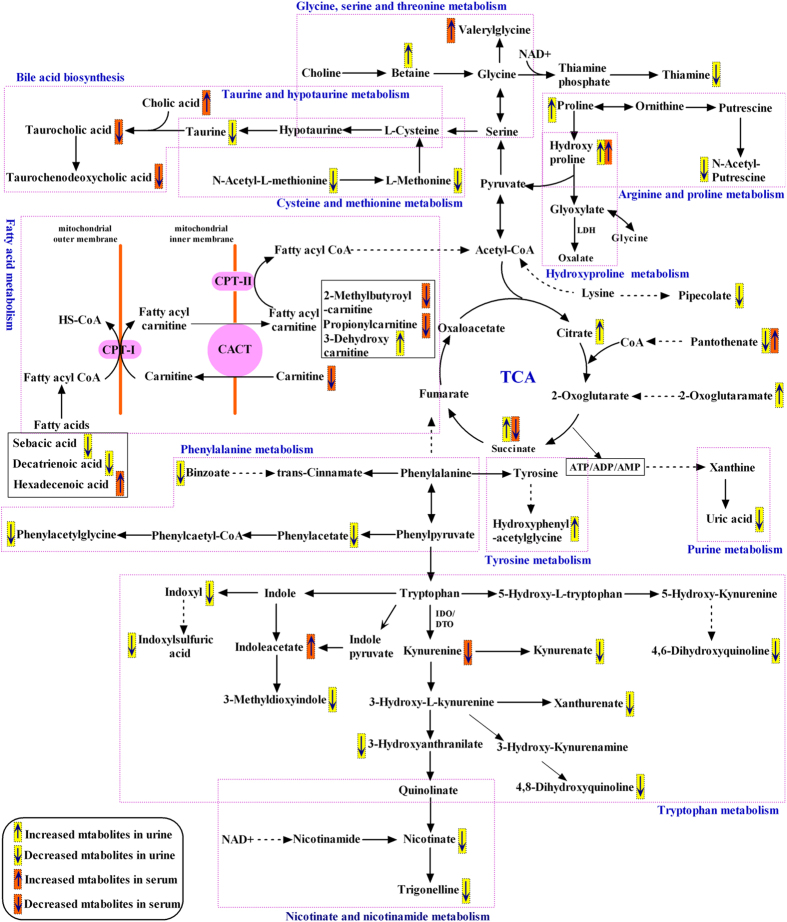
Metabolic pathways related to the differential metabolites identified in the HLP induced crystal kidney injury.

In this study, we successfully constructed HLP-induced crystal renal injury model in SD rats by orally administered 5% HLP^15^. UPLC-Q-TOF/MS was applied to profile the abundances of metabolites of urine and serum at different time points in the control and HLP-treated rats. 42 differentially expressed urinary metabolites and 13 differentially expressed serum metabolites were identified. The summary of pathway analysis based on IPA analysis using the MetaboAnalyst platform is shown in Figure S4. According to KEGG pathway database (http://www.genome.jp/kegg/), we constructed a metabolic pathway network based on the differential metabolites (Fig. 5). From this network, the differential metabolites are mainly related to several metabolic pathways, such as amino acid metabolism, taurine and hypotaurine metabolism, bile acid synthesis, energy metabolism, TCA cycle, purine metabolism, vitamin metabolism, and nicotinic acid and nicotinamide metabolism.

Betaine is catabolized from Choline via a series of enzyme reactions that occur mainly in the mitochondria of liver and kidney cells^17^. Activity of Choline and Betaine is considered an important methyl donor mitochondrial protein and nucleic acid synthesis required^18^, involved in methionine cycle. In addition, betaine, as an organic osmotic regulator, can effectively protect cells, proteins and enzymes from the external environment stress, which plays an important role on the mechanism of osmotic adjustment for a variety of kidney cells. Betaine synthesized in the kidney, but mainly in the cortex and outer medulla, thus accumulated betaine synthesis and osmotic regulation were occurred in different parts of the kidney, and studies show that Betaine metabolism in kidney cells are extracellular permeability of the adjust. In the present study, betaine increased significantly in both RP mode and HILIC mode for all groups of HLP intervention, indicating that betaine metabolism has been severely disrupted and thus further impact the osmosis of the kidney during the process of HLP-induced CaOx crystal kidney injury.

Taurine is an important sulfur-containing amino acid, and its balance in the body relies on multiple biological processes, including food intake, endogenous synthesis, and kidney reabsorption^19^. Its endogenous synthesis is mainly through the cysteine and methionine metabolism. Methionine produce cysteine by transculturation; and cysteine synthesized hypotaurine by cysteine deoxygenase (CD) and cysteine sulfinate decarboxylase (CSD). Hypotaurine with unstable chemical properties is easily oxidized to generate taurine. Some of taurine are reabsorbed through taurine transporter in kidneys, the first place of taurine regulation; and the others are discharged through the urine^20^. Taurine affect various physiological function of the kidney. In the renal medulla part, it can be as an important non-ionic permeability material to ensure kidney osmoregulation and cell volume regulation^21^. In the glomerular area, it plays a role in renal protection by scavenging reactive oxygen species (ROS)^22^. It has been reported taurine has a protective effect on CaOx crystal kidney injury in rats. It can reduce the kidney injury induced by calcium oxalate stones through a decrease of mitochondrial membrane damage and an increase of SOD and GSH-Px^23^. In the present study, taurine in the urine significantly reduced after the second week of HLP treated rats. This result is in consistence with our previous results^16^. Clinical studies also showed urinary taurine has a significant decline in patients with CaOx stones. In addition, taurine is involved in the conjugation of bile salts in the liver by converting primary bile salts into secondary bile salts. After synthesis from cholesterol in the liver, cholic acid, an important primary bile acid, are conjugated with taurine and/or glycine, before secretion into the bile canaliculi and the small intestine^24^. The bile acids serve as an important physiological function in regulation of lipid metabolism^25^. Several studies^26^,^27^,^28^ reported that the intestinal flora plays an important role on regulation of bile acid metabolism. In this study, cholic acid increased significantly in the blood of the HLP-treated rats, while two secondary bile acids, taurocholic acid and taurodeoxycholic acid, decreased significantly, indicating that bile acid metabolism as well as lipid metabolism have been significantly affected during HLP-induced crystal renal. It has been reported that conjugated bile acids may effectively reduce oxalate excretion in urine, thus reduce the risk of the formation of calcium oxalate stones^29^. In our study, it is possible that significant reduction of serum taurine-conjugated secondary bile salts play a role in promoting the excretion of oxalate.

Carnitine is a hydrophilic quaternary ammonium, essential for fatty acid β-oxidation, primarily by carrying long-chain fatty acids across the mitochondrial membrane into the activity of the mitochondrial mechanisms^30^. Carnitine in the liver and kidney endogenous synthesis^31^,^32^ and not through the metabolism of the prototype excreted into the urine^33^. The kidney plays an important role in carnitine homeostasis *in vivo*. Under normal physiological conditions, substantially, all the filtered carnitine was reabsorbed by renal tubules through an active carnitine transport mechanism^34^,^35^. Carnitine synthetic defect has little effect on the stability of carnitine, in contrast, carnitine transport barriers will lead to severe carnitine deficiency, which affects β oxidation of fatty acids^33^. Propionyl-L-carnitine, an endogenous short chain fatty acyl carnitine, after both carnitine and acetyl carnitine, is an important constituent of the carnitine pool^36^. *In vitro* experiments showed, propionyl- L-carnitine is mainly metabolized to carnitine and acetyl-L-carnitine in the kidney, and 95% of propionyl- L-carnitine is reabsorbed in the tubules^37^. In addition to the β oxide storage material increase as carnitine mitochondria, propionyl-L-carnitine may also reduce the Nox4 mediated oxidative stress and endothelial dysfunction^38^. In the present study, we observed a significant increase of KIM-1 levels in urine and serum after HLP treatment, indicating HLP-induced calcium oxalate crystal causes significant kidney epithelial cell damage, thus affecting the transportation of carnitine and acyl carnitine. This may cause a significant decrease of carnitine and propionyl-L-carnitine in blood in HLP-treated rats, further affect β oxidation of fatty acids, resulting in dysfunction of energy metabolism. In addition, we observed that citric acid (isocitrate) and succinic acid in the urine significantly increased, while succinic acid in the blood significantly decreased, in the HLP-treated rats. These results suggested that the Krebs cycle and mitochondrial function are disturbed during the formation of HLP-induced calcium oxalate crystal, thus resulting in the abnormalities of body’s energy metabolism.

Several amino acids and their metabolites significantly changed in both blood and urine of the HLP-treated rats. They are mainly involved in arginine and proline metabolism, phenylalanine metabolism, tyrosine metabolism, tryptophan metabolism, cysteine and methionine metabolisms, especially tryptophan metabolism In urine, nine tryptophan metabolism-related metabolites including indoxyl, 3-Hydroxyanthranilic acid, 4,6-dihydroxyquinoline, 4,8-dihydroxyquinoline, 3-methyldioxyindole, indoleacrylic acid, kynurenic acid, indoxylsulfuric acid, and xanthurenic acid significantly down-regualted, while in serum, indolelactic acid significantly up-regulated and kynurenine significantly down-regulated. Tryptophan is an important essential amino acid in the body, and mainly involved in metabolic pathways including protein synthesis, serotonin pathway and kynurenine pathway^39^. About 95% of tryptophan in the body degraded by kynurenine metabolic pathway^40^, and a small amount of tryptophan, which is not absorbed in the intestine, degraded by the intestinal flora to produce indole through indole metabolisms^41^. The rate-limiting enzymes of the conversion of tryptophan to kynurenine are 2,3-dioxygenase (TDO) and indoleamine 2,3-dioxygenase (IDO)^42^. TDO mainly expressed in liver tissues, and IDO is widely distributed in tissues other than liver tissue^43^. Therefore, blood kynurenine/tryptophan ratio is often used as IDO activity indicator^44^. In the present study, the blood kynurenine reduced significantly at the fourth week of HLP treatment, while the blood kynurenine/tryptophan ratio had no significant changes in the HLP-treated rats. These results suggested that the effect of HLP on kynurenine may be not related to the activity of IDO. Indole compounds produced by indole metabolisms and kynurenine metabolic compounds including kynurenine, kynurenic acid, anthranilic acid, 3-hydroxykynurenine, 3-hydroxyanthranilic acid, and quinolinic acid are considered as uremic toxins, which are closely related to the worsening renal function^39^. The role of kidney on the metabolism of tryptophan is not only involved in metabolism of kynurenine, but also involved in the clearance of related metabolites. We observed that indoles including indoxyl, indoxylsulfuric acid and kynurenine metabolites including 3-hydroxyanthranilic acid, kynurenic acid, significantly decreased in the urine of HLP-treated rats, indicating that HLP-induced crystal kidney injury may affect the clearance of the above uremic toxins.

In this study, we observed that vitamins B and its metabolites in urine, such as thiamine (VB1), riboflavin (VB2), nicotinic acid (VB3), trigonelline, and pantothenic acid (VB5), significantly decreased in HLP-treated rats, compare to the control rats. Vitamin B, a class of water-soluble vitamins, acts as a coenzyme to be involved in the body’s energy metabolism including carbohydrates, fatty acids, and amino acid metabolisms. VB1, as a cofactor thiamin diphosphate, may catalyze glucose metabolism, including the decarboxylation and transketolase effect of α- keto acid. VB2 acts as FAD and FMN to participate in electronic form of transmission and chain fatty acid oxidation. VB3 is necessary for the synthesis of NADH substances and provide protons during oxidative phosphorylation. VB5, as an important precursor of coenzyme A, is involved in glucose metabolism, citric acid cycle, fatty acids and cholesterol analysis, and other metabolic processes^18^,^45^. Water soluble Vitamin C, as the electron donor, plays a role in anti-oxidation by directly scavenging reactive oxygen species, thus prevents kidney epithelial cells from free radical damage and prevent the formation of calcium oxalate crystals^46^. And Vitamin C can decompose and metabolize to generate oxalate in the body, and excessive VC can induce hyperoxaluria and stone formation^47^. Kidney is involved in the metabolism and distribution of vitamins^48^,^49^,^50^. It has been reported that chronic kidney disease affects the metabolism and excretion of multivitamins. Our results indicated that HLP-induced kidney injury interferes with the homeostasis of VB. In addition, Pantothenic Acid increased significantly in the blood of HLP treated rats, indicating that HLP-induced renal injury may influence pantothenic acid, an important precursor of coenzyme A, thereby affecting mitochondrial function. Ascorbate 2-sulfate, as sulfate conjugates of ascorbate, excretes in urine. Urinary excretion of ascorbate 2-sulfate significantly decreased in HLP-treated rats, compared to the control group, which may increase the conversion of ascorbate to oxalate.

## Conclusion

In this study, we combined RP and HILIC with mass spectrometry to analyze endogenous metabolites in favor of a comprehensive analysis of a more systematic, and mutual authentication modes to improve the detection of credibility. Using multivariate statistical analysis by OPLS-DA and univariate Wilcoxon Mann-Whitney Test, we selected 42 differential metabolites in the urine, and 13 differential metabolites in the blood. Metabolic pathways are identified to be involved in amino acid metabolism, taurine times taurine metabolism, bile acid synthesis, energy metabolism, TCA cycle, purine metabolism, vitamin metabolism, nicotinic acid and nicotinamide metabolism. This study suggested that a number of metabolic pathways are dysfunctional in the HLP-induced crystal kidney injury, and further studies on those pathways are warranted to better understand the metabolic mechanism of CaOx nephrolithiasis.

## Materials and Methods

### Chemicals and reagents

Methanol (HPLC grade) and acetonitrile (HPLC grade) were purchased from Merck (Darmstadt, Germany). Formic acid (FA) (HPLC grade) was purchased from the Fluka Chemical Corp (Buchs, Switzerland). 2-Chloro-L-phenylalanine (as an internal standard) was purchased from Sigma-Aldrich Company (St Louis, MO, USA). Hydroxyproline (HLP) (99% purity) was purchased from GL Biochem Ltd. (Shanghai, China). Sodium carboxymethylcellulose (CMC-Na) was purchased from Sinopharm Chemical Reagent Co., Ltd. (Shanghai, China). Chromatography Mass prepared by the Milli-Q system (Millipore, Bedford, MA, USA) with ultrapure water. Kidney injury molecule-1 (KIM-1) ELISA kit was purchased from Wuhan Uscn Life Science, Inc. (Wuhan, China). Von Kossa kit was purchased from GENMED Scientific Inc. (Shanghai, China). Hydroxyproline (300 mg/mL) was dissolved into 0.5% CMC-Na and stored at 4 °C.

### Experimental animals

All animal studies were performed in accordance with the National Institutes of Health (NIH) guide for the Care and Use of Laboratory Animals. The experimental procedures were approved by the Ethical Committee for the Experimental Use of Animals at Second Military Medical University (Shanghai, China). Thirty-one Sprague-Dawley (SD) rats (120–130 g) at the age of 5 weeks were purchased from Shanghai SLAC Laboratory Animal Co., Ltd (Shanghai, China). Three rats per cage were maintained under standard laboratory conditions (temperature of 20–25 °C, relative humidity of 55–65%, and 12 h/12 h light/dark cycle) with aseptic food and tap water ad libitum. After one week of habituation, rats were randomized according to the weight into the control group (C), 7-day HLP model group (H-7d), 14-day HLP model group (H-14d), 21-day HLP model group (H-21d), and 28-day HLP model group (H-28d). Each group has 6 rats except HLP -28D model group with seven rats. The model group was given 5 g/kg dose of hydroxyproline (HLP) per day; the control group received the same volume of 0.5% CMC-Na. Body weight was recorded once every three days. Before starting modeling, the control group rats were placed in metabolic cages to collect 24 hours urine, orbital blood, and then normal feeding. In the 7 days, 14 days and 21 days, the C group and H each group of rats were placed in metabolic cages to collect 24 h urine during normal feeding, drinking, and recording the volume of urine. After collecting urine, orbital blood was collected. Thereafter, the C group back to continue normal feeding, the rats were H group after cardiac perfusion, collecting both kidneys. After removing the capsule and pelvis, one kidney was quickly placed in −80 °C to save; the other side of kidney was in 4% paraformaldehyde fixed. After 28 days of modeling, the urine, blood and kidney samples both in H-28d group and C group were collected.

### Sample collection and preparation

The urine was collected, immediately centrifuged at 4000 rpm for 5 min, the supernatant was saved. The blood samples were placed at room temperature for one hour, and serum was extracted by centrifugation at 4000 rpm, 4 °C for 5 min. The urine and serum were both immediately stored at −80 °C before UHPLC-Q-TOF/MS analysis.

### Histopathological examination

The kidney tissue was fully immobilized by placing in 4% paraformaldehyde, and fixed tissues were processed routinely for paraffin embedding, and 3–4 μm sections were prepared and stained with von Kossa, according to the instructions of the von Kossa kit. The calcium deposition was estimated in various parts of the observation of renal tissue.

### Biochemical analysis

Each group was measured in urine calcium, phosphorus, magnesium-creatinine ratio, as well as fresh renal tissue calcium content, respectively. Urinary calcium (Ca), magnesium (Mg) and phosphorus (P) were determined in Nanjing Jiancheng Bioengineering Institute (Nanjing, China) via colorimetry with Methylthymol blue for Ca, Arsenazo i for Mg. KIM-1 is not only a good biological indicator of acute kidney injury, but also as a functional molecule to participate in the process of renal tubular damage and repair^51^. The levels of serum and urinary KIM-1 were analyzed using commercially available Enzyme-linked Immunosorbent Assays (ELISA) according to manufacturer’s protocol.

### UHPLC-Q-TOF MS analysis

The frozen urine and serum samples were thawed at room temperature. 300 μL of the methanol (containing 25 μg/ml 2-Chloro-L-phenylalanine as the internal standard) was added into 100 μL urine or serum samples to precipitate the protein and extract the metabolites, and mixed by vortex 5 min. The mixed samples was placed at RT for 10 min and centrifuged at 13,000 rpm, 4 °C for 15 min, and the clear supernatant was transferred to a sampler vial. An in-house quality control (QC) was prepared by pooling and mixing the same volume of each sample. The QC sample was run six times prior to the start of the analytical run to “condition” the system and analyzed after every 9 samples to check for system stability.

UHPLC-Q-TOF/MS analysis was performed using an Agilent 1290 Infinity LC system coupled to an Agilent 6538 Accurate-Mass Quadrupole Time-of-Flight (Q-TOF) mass spectrometer (Agilent, USA). RP chromatographic separations were performed at 40 °C using a Waters XBridge^TM^ BEH C18 analytical column (2.1 mm × 100 mm, 2.5 μm, Waters, Milford, MA). The flow rate was 0.4 mL/min and the injection volume was 3 μL.The mobile phase consisted of 0.1% formic acid (A) and ACN modified with 0.1% formic acid (B). The gradient elution conditions for urinary samples were 5% B at 0–2 min, 5–15% B at 2–10 min, 15–30% B at 10–14 min, 30–95% B at 14–17 min, 95% B at 17–19 min and followed by re-equilibrted step of 6 min.As for serum samples, the following gradient program was used: 5%B at 0–2 min, 5–95%B at 2–17 min, 95%B at 17–19 min. HILIC separations of urinary samples were performed at 25 °C on an Acquity UPLC BEH HILIC column (2.1 mm × 100 mm, 1.7 μm, Waters, Milford, MA). The mobile phase consisted of 10 mM ammonium formate modified with 0.1% formic acid(A) and ACN modified with 0.1% formic acid (B). The flow rate was 0.4 mL/min and the injection volume was 3 μL. The total run time for one sample was 20 min including 5 min for equilibration. The optimized UPLC elution conditions were 95%B at 0–5 min, 95–87%B at 5–6 min, 87–82%B at 6–12 min, 82–75%B at 12–15 min.

Mass spectrometry was operated in both positive and negative ion modes. The capillary voltage was 4 kV in positive mode and 3.5 kV in negative mode, the drying gas flow was 11 L/min, and the gas temperature was 350 °C. The nebulizer pressure was set at 45 psig. The fragmentor voltage was set at 120 V and skimmer voltage was set at 60 V. All analyses were conducted using a mixture of 0.5 mM purine (m/z 121.0508 in positive mode; m/z 119.03632 in negative mode) and 0.5 mM hexakis phosphazine (m/z 922.0098 in positive mode; m/z 966.000725 in negative mode) as internal standards to ensure mass accuracy and reproducibility. Data were collected in a centroid mode and the mass range was set at m/z 50–1100 using an extended dynamic range. MS/MS analysis was carried out to study the structure of the potential biomarkers and the collision energy was range from 10 to 40 eV.

### Data processing and statistical analysis

The raw data in the instrument specific format (.d) were collected and converted into a common (mz.data) format using the Agilent Masshunter Qualitative Analysis B.04.00 software (Agilent Technologies, USA), in which the filtration threshold of the high of the absolute peak was set to 500, and the isotope interferences were excluded. In the R software platform, the XCMS program was used to identify a peak, retention time correction, automatic integration pretreatment^52^, to give a containing sample name, m/z-RT pair, the peak area of the visualization matrix. The parameter of the XCMS program was set as following: full width at half maximum (FWHM) = 8, S/N (snthresh) = 5, the bandwidth (bw) = 10. After 80% based on the principle of selection, frequency of more than 80% of the ions present in each group retained samples^53^, and fill in missing values averaged. Then all the ions within the molecular ion peak of the standard substance (positive ion mode: 200.0473; negative ion mode: 198.0328) as a standard ion normalized. Calculation sequence QC sample (n = 7) of each feature RSD (%) value, the larger the variation, there may occasionally related in QC samples^54^. Screened RSD values less than 20% of the features, subsequent chemometric analysis.

Pattern recognition analysis was performed by importing the pretreatment data into SIMCA-P 13.0 Demo (Umetrics, Umea, Sweden). Prior to multivariate analysis, the resultant data matrices were mean-centered and scaled to Pareto variance, and unsupervised principal component analysis (PCA) was used to observe the separating trends and metabolic trajectories between each set of samples, and eliminate outliers. To identify a metabolite candidate that could differentiate HLP treated rats from control rats at each time point, orthogonal partial least squares discriminate analysis (OPLS-DA) was performed. Variable importance plot (VIP) for the confirmation of the importance or power of the selected candidates was used to select metabolites candidates with the threshold value of 1. In addition, the model of OPLS-DA was evaluated according to the cross-validation of R2, Q2 value and p-value form cross-validation analysis of variance (CV-ANOVA), and the p value threshold was set at 0.05. Statistically significant differences in mean values were tested using SPSS 17.0 for an independent sample *t*-test and *p* < 0.05 was considered statistically significant. For significantly differential metabolites, metabolic pathway analysis (MetPA) and Heatmap analysis were performed using MetaboAnalyst platform (http://www.metaboanalyst.ca).

## Additional Information

**How to cite this article**: Gao, S. *et al.* Metabolomics analysis for hydroxy-L-proline-induced calcium oxalate nephrolithiasis in rats based on ultra-high performance liquid chromatography quadrupole time-of-flight mass spectrometry. *Sci. Rep.* **6**, 30142; doi: 10.1038/srep30142 (2016).

## Supplementary Material

Supplementary Information

## Figures and Tables

**Table 1:** Significantly differential metabolites in urine of rats with HLP-induced crystal renal injury.

No.	Metabolites	Formula	^a^FC(D7)	FC(D14)	FC(D21)	FC(D28)	Related pathway
1	L-Proline	C5H9NO2	7.94^†^	5.17^†^	12.53^‡^	14.59^‡^	Arginine and proline metabolism
2	Betaine	C5H11NO2	21.64^†^	13.11^‡^	12.42^‡^	9.17^‡^	Glycine, serine and threonine metabolism
3	Benzoic acid	C7H6O2	0.52^*^	0.26^*^	0.42^*^	—	Phenylalanine metabolism
4	Taurine	C2H7NO3S	—	0.54^†^	0.5^†^	0.43^†^	Taurine and hypotaurine metabolism
5	Nicotinic acid	6H5NO2	0.48^†^	0.35^‡^	0.47^‡^	0.41^†^	Nicotinate and nicotinamide metabolism
6	Maltol	C6H6O3	0.45	0.54^†^	—	0.37^*^	Polyketide sugar unit biosynthesis
7	L-Pipecolic acid	C6H11NO2	0.53^‡^	—	0.51^‡^	0.42^‡^	Lysine degradation
8	N-Acetylputrescine	C6H14N2O	0.41^‡^	0.28^‡^	0.38^†^	0.25^†^	Arginine and proline metabolism
9	Indoxyl	C8H7NO	0.3^*^	0.21^†^	0.26^†^	—	Tryptophan metabolism
10	Phenylacetic acid	C8H8O2	—	—	—	0.43^*^	Phenylalanine metabolism
11	Trigonelline	C7H7NO2	0.4^‡^	0.27^‡^	0.41^‡^	0.44^‡^	Nicotinate and nicotinamide metabolism
12	2/4-Oxoglutaramate	C5H7NO4	7.2^*^	16.99^*^	19.12^†^	21.33^‡^	—
13	Proline betaine	C7H13NO2	0.55^‡^	0.46^‡^	0.61^†^	0.54^‡^	—
14	3-Dehydroxycarnitine	C7H15NO2	2.17^†^	—	—	—	Energy metabolism
15	L-Methionine	C5H11NO2S	0.39^†^	0.42^†^	—	—	Cysteine and methionine metabolism
16	3-Hydroxyanthranilic acid	C7H7NO3	0.25^†^	0.44^*^	0.6^*^	—	—
17	Indole-3-carboxylic acid	C9H7NO2	—	0.36	0.46^†^	0.41^*^	Tryptophan metabolism
18	2-Indolecarboxylic acid	C9H9NO2	0.24^†^	0.13^‡^	0.42^†^	0.13^†^	Tryptophan metabolism
19	3-Methyldioxyindole	C4H6O4	—	0.39^*^	—	—	Tryptophan metabolism
20	Succinic acid	C6H7N5O	39.64^†^	50.23^‡^	43.83^†^	45.49^‡^	TCA
21	7-Methylguanine	C5H4N4O3	0.48^†^	0.46^*^	0.71^*^	0.49^*^	—
22	Uric acid	C10H14O2	0.57^*^	—	—	0.45^*^	Purine metabolism
23	Decatrienoic acid	C6H6O4S	—	0.36^†^	—	—	Energy metabolism
24	Phenol sulfate	C11H9NO2	—	—	0.09^*^	—	—
25	Indoleacrylic acid	C7H8O4S	—	—	—	0.42^*^	Tryptophan metabolism
26	p-Cresol sulfate	C10H7NO3	—	—	0.06^*^	—	—
27	Kynurenic acid	C6H6O5S	—	0.41^*^	0.39^*^	0.31^*^	Tryptophan metabolism
28	Pyrocatechol sulfate	C7H13NO3S	0.4^*^	—	0.11^†^	—	—
29	N-Acetyl-L-methionine	C6H8O7	0.15^†^	0.37^*^	0.31^†^	0.31^*^	Cysteine and methionine metabolism
30	Citric acid	C10H11NO3	—	—	—	2.39^*^	TCA
31	Phenylacetylglycine	C10H18O4	—	—	0.32^*^	—	Phenylalanine metabolism
32	Sebacic acid	C8H18N4O2	0.41^*^	—	0.5^*^	0.45^*^	Energy metabolism
33	ADMA	C10H7NO4	0.38^†^	—	1.47^†^	2.11^‡^	Arginine metabolism
34	Xanthurenic acid	C10H11NO4	0.66	0.4^*^	0.42^†^	0.19^†^	Tryptophan metabolism
35	Hydroxyphenylacetylglycine	C10H16N2O3	1.48^*^	—	—	2.02^*^	Tyrosine metabolism
36	L-prolyl-L-proline	C8H7NO4S	0.4^‡^	0.31^†^	0.45^†^	0.39^*^	—
37	Indoxylsulfuric acid	C10H13NO4	0.43^*^	0.38^*^	0.23^†^	—	Tryptophan metabolism
38	3-Methoxytyrosine	C9H17NO5	0.5^‡^	0.48^†^	0.21^‡^	0.15^‡^	—
39	Pantothenic Acid	C9H17NO6	0.49^†^	0.72^*^	—	0.6^*^	Vitamin B metabolism
40	Ascorbate 2-sulfate	C6H8O9S	—	—	0.41^†^	0.4^†^	vitamin C metabolism
41	Thiamine	C12H16N4OS	—	0.43^†^	—	—	Vitamin B metabolism
42	Riboflavin (VB2)	C17H20N4O6	0.26^*^	0.19^‡^	—	0.4^*^	Vitamin B metabolism

^a^FC, fold changes at different time points, compared to the control rats.^*^p < 0.05, ^†^p < 0.001, ^‡^p < 0.001,“—”no statistically significant changes.

**Table 2:** Significantly differential metabolites in serum of rats with HLP-induced crystal renal injury.

No.	Metabolites	Formula	^a^FC(D7)	FC(D14)	FC(D21)	FC(D28)	Related pathway
1	Succinic acid	C4H6O4	—	0.64^*^	—	0.44^†^	TCA cycle
2	4-Hydroxy-L-proline	C5H9NO3	1.62^†^	3.43^†^	1.79^†^	1.62^†^	—
3	Creatine	C4H9N3O2	2.86^‡^	6.98^*^	3.28^‡^	2.76^†^	
4	Valerylglycine	C7H13NO3	2.09^*^	—	2.02^†^	2.45^*^	fatty acid beta-oxidation
5	L-Carnitine	C7H15NO3	—	0.68^†^	0.54^†^	0.37^†^	Energy metabolism
6	Indolelactic acid	C11H11NO3	—	5.47^†^	1.99^*^	1.86^†^	Tryptophan metabolism
7	Kynurenine	C10H12N2O3	—	—	—	0.5^*^	Tryptophan metabolism
8	Pantothenic Acid	C9H17NO5	1.8^†^	—	2.01^†^	1.56	Vitamin B metabolism
9	Propionylcarnitine	C10H19NO4	—	0.65^†^	0.47^†^	0.43^†^	fatty acid beta-oxidation
10	2-Methylbutyroylcarnitine	C12H23NO4	—	0.64^†^	0.58^*^	0.33^*^	fatty acid metabolism
11	Hexadecenoic Acid	C16H30O2	3.43^†^	—	—	—	fatty acid metabolism
12	Cholic acid	C24H40O5	—	6.25^*^	—	4.6^*^	—
13	Tauroursodeoxycholic acid	C26H45NO6S	—	0.4^†^	0.36^†^	—	—
14	Taurocholic acid	C26H45NO7S	—	0.39^†^	0.52^*^	—	Taurine and hypotaurine metabolism

^a^FC, fold changes at different time points, compared to the control rats. ^*^p < 0.05, ^†^p < 0.001, ^‡^p < 0.001,“—”no statistically significant changes.

## References

[b1] Rule A. D., Bergstralh E. J., Melton L. J. 3rd, Li X., Weaver A. L. & Lieske J. C. Kidney stones and the risk for chronic kidney disease. Clin J Am Soc Nephrol 4, 804–811 (2009). 19339425 10.2215/CJN.05811108 PMC2666438

[b2] El-Zoghby Z. M. *et al.* Urolithiasis and the risk of ESRD. Clin J Am Soc Nephrol 7, 1409–1415 (2012). 22745275 10.2215/CJN.03210312 PMC3430957

[b3] Pak C. Y. Etiology and treatment of urolithiasis. Am. J. Kidney Dis. 18, 624–637 (1991). 1962646 10.1016/s0272-6386(12)80602-0

[b4] Kok D. J. & Khan S. R. Calcium oxalate nephrolithiasis, a free or fixed particle disease. Kidney Int. 46, 847–854 (1994). 7996806 10.1038/ki.1994.341

[b5] Tasca A. Metabolic syndrome and bariatric surgery in stone disease etiology. Curr Opin Urol 21, 129–133 (2011). 21191301 10.1097/MOU.0b013e3283435cbc

[b6] Mohammadjafari H., Barzin M., Salehifar E., Khademi K. M., Aalaee A. & Mohammadjafari R. Etiologic and epidemiologic pattern of urolithiasis in north iran;review of 10-year findings. Iran J Pediatr 24, 69–74 (2014). 25793048 PMC4359607

[b7] Coe F. L., Parks J. H. & Asplin J. R. The pathogenesis and treatment of kidney stones. N. Engl. J. Med. 327, 1141–1152 (1992). 1528210 10.1056/NEJM199210153271607

[b8] Khan S. R. Animal models of kidney stone formation: an analysis. World J Urol 15, 236–243 (1997). 9280052 10.1007/BF01367661

[b9] Khan S. R. Experimental calcium oxalate nephrolithiasis and the formation of human urinary stones. Scanning microscopy 9, 89–100, discussion 100-101 (1995). 8553028

[b10] de Water R. *et al.* Experimental nephrolithiasis in rats: the effect of ethylene glycol and vitamin D3 on the induction of renal calcium oxalate crystals. Scanning microscopy 10, 591–601, discussion 601-603 (1996). 9813634

[b11] Robinson M., Pond C. L., Laurie R. D., Bercz J. P., Henningsen G. & Condie L. W. Subacute and subchronic toxicity of ethylene glycol administered in drinking water to Sprague-Dawley rats. Drug Chem Toxicol 13, 43–70 (1990). 2379473 10.3109/01480549009011069

[b12] Poldelski V., Johnson A., Wright S., Rosa V. D. & Zager R. A. Ethylene glycol-mediated tubular injury: identification of critical metabolites and injury pathways. Am. J. Kidney Dis. 38, 339–348 (2001). 11479160 10.1053/ajkd.2001.26099

[b13] Knight J., Jiang J., Assimos D. G. & Holmes R. P. Hydroxyproline ingestion and urinary oxalate and glycolate excretion. Kidney Int. 70, 1929–1934 (2006). 17021603 10.1038/sj.ki.5001906 PMC2268952

[b14] Knight J. & Holmes R. P. Mitochondrial hydroxyproline metabolism: implications for primary hyperoxaluria. Am. J. Nephrol. 25, 171–175 (2005). 15849464 10.1159/000085409 PMC4756647

[b15] Khan S. R., Glenton P. A. & Byer K. J. Modeling of hyperoxaluric calcium oxalate nephrolithiasis: experimental induction of hyperoxaluria by hydroxy-L-proline. Kidney Int. 70, 914–923 (2006). 16850024 10.1038/sj.ki.5001699

[b16] Gao S. *et al.* Urinary metabonomics elucidate the therapeutic mechanism of Orthosiphon stamineus in mouse crystal-induced kidney injury. J Ethnopharmacol 166, 323–332 (2015). 25794803 10.1016/j.jep.2015.03.025

[b17] Craig S. A. Betaine in human nutrition. Am. J. Clin. Nutr. 80, 539–549 (2004). 15321791 10.1093/ajcn/80.3.539

[b18] Depeint F., Bruce W. R., Shangari N., Mehta R. & O’Brien P. J. Mitochondrial function and toxicity: role of B vitamins on the one-carbon transfer pathways. Chem. Biol. Interact. 163, 113–132 (2006). 16814759 10.1016/j.cbi.2006.05.010

[b19] Terrill J. R., Grounds M. D. & Arthur P. G. Taurine deficiency, synthesis and transport in the mdx mouse model for Duchenne Muscular Dystrophy. Int. J. Biochem. Cell Biol. 66, 141–148 (2015). 26239309 10.1016/j.biocel.2015.07.016

[b20] Lambert I. H., Kristensen D. M., Holm J. B. & Mortensen O. H. Physiological role of taurine--from organism to organelle. Acta physiologica (Oxford, England) 213, 191–212 (2015). 10.1111/apha.12365 25142161

[b21] Burg M. B. Molecular basis of osmotic regulation. Am. J. Physiol. 268, F983–F996 (1995). 7611465 10.1152/ajprenal.1995.268.6.F983

[b22] Jeon S. H. *et al.* Taurine reduces FK506-induced generation of ROS and activation of JNK and Bax in Madin Darby canine kidney cells. Hum Exp Toxicol 29, 627–633 (2010). 20056734 10.1177/0960327109359019

[b23] Li C. Y., Deng Y. L. & Sun B. H. Taurine protected kidney from oxidative injury through mitochondrial-linked pathway in a rat model of nephrolithiasis. Urol. Res. 37, 211–220 (2009). 19513707 10.1007/s00240-009-0197-1

[b24] Hickman M. A., Morris J. G. & Rogers Q. R. Intestinal taurine and the enterohepatic circulation of taurocholic acid in the cat. Adv. Exp. Med. Biol. 315, 45–54 (1992). 1509964 10.1007/978-1-4615-3436-5_6

[b25] Chiang J. Y. Bile acids: regulation of synthesis. J. Lipid Res. 50, 1955–1966 (2009). 19346330 10.1194/jlr.R900010-JLR200 PMC2739756

[b26] Swann J. R. *et al.* Systemic gut microbial modulation of bile acid metabolism in host tissue compartments. Proc. Natl. Acad. Sci. USA 108 Suppl 1, 4523–4530 (2011). 20837534 10.1073/pnas.1006734107 PMC3063584

[b27] David L. A. *et al.* Diet rapidly and reproducibly alters the human gut microbiome. Nature 505, 559–563 (2014). 24336217 10.1038/nature12820 PMC3957428

[b28] Qi Y. *et al.* Bile acid signaling in lipid metabolism: metabolomic and lipidomic analysis of lipid and bile acid markers linked to anti-obesity and anti-diabetes in mice. Biochim. Biophys. Acta 1851, 19–29 (2015). 24796972 10.1016/j.bbalip.2014.04.008 PMC4219936

[b29] Emmett M. *et al.* Conjugated bile acid replacement therapy reduces urinary oxalate excretion in short bowel syndrome. Am. J. Kidney Dis. 41, 230–237 (2003). 12500242 10.1053/ajkd.2003.50012

[b30] Bremer J. Carnitine–metabolism and functions. Physiol. Rev. 63, 1420–1480 (1983). 6361812 10.1152/physrev.1983.63.4.1420

[b31] Rebouche C. J. & Engel A. G. Tissue distribution of carnitine biosynthetic enzymes in man. Biochim. Biophys. Acta 630, 22–29 (1980). 6770910 10.1016/0304-4165(80)90133-6

[b32] Rebouche C. J. Carnitine function and requirements during the life cycle. FASEB J. 6, 3379–3386 (1992). 1464372

[b33] El-Hattab A. W. & Scaglia F. Disorders of carnitine biosynthesis and transport. Mol. Genet. Metab. 116, 107–112 (2015). 26385306 10.1016/j.ymgme.2015.09.004

[b34] Brooks D. E. & McIntosh J. E. Turnover of carnitine by rat tissues. Biochem. J. 148, 439–445 (1975). 1200987 10.1042/bj1480439 PMC1165561

[b35] Huang W. *et al.* Carnitine transport and its inhibition by sulfonylureas in human kidney proximal tubular epithelial cells. Biochem. Pharmacol. 58, 1361–1370 (1999). 10487540 10.1016/s0006-2952(99)00219-1

[b36] Pace S., Longo A., Toon S., Rolan P. & Evans A. M. Pharmacokinetics of propionyl-L-carnitine in humans: evidence for saturable tubular reabsorption. Br J Clin Pharmacol 50, 441–448 (2000). 11069438 10.1046/j.1365-2125.2000.00280.x PMC2014409

[b37] Evans A. M., Mancinelli A. & Longo A. Excretion and metabolism of propionyl-L-carnitine in the isolated perfused rat kidney. J. Pharmacol. Exp. Ther. 281, 1071–1076 (1997). 9190838

[b38] Scioli M. G. *et al.* Propionyl-L-Carnitine Enhances Wound Healing and Counteracts Microvascular Endothelial Cell Dysfunction. PLoS One 10, e0140697 (2015). 26473356 10.1371/journal.pone.0140697 PMC4608702

[b39] Sallee M., Dou L., Cerini C., Poitevin S., Brunet P. & Burtey S. The aryl hydrocarbon receptor-activating effect of uremic toxins from tryptophan metabolism: a new concept to understand cardiovascular complications of chronic kidney disease. Toxins (Basel) 6, 934–949 (2014). 24599232 10.3390/toxins6030934 PMC3968369

[b40] Pawlak D., Tankiewicz A., Mysliwiec P. & Buczko W. Tryptophan metabolism via the kynurenine pathway in experimental chronic renal failure. Nephron 90, 328–335 (2002). 11867954 10.1159/000049069

[b41] Aronov P. A. *et al.* Colonic contribution to uremic solutes. J. Am. Soc. Nephrol. 22, 1769–1776 (2011). 21784895 10.1681/ASN.2010121220 PMC3171947

[b42] Adam R. *et al.* Role of human brain microvascular endothelial cells during central nervous system infection. Significance of indoleamine 2,3-dioxygenase in antimicrobial defence and immunoregulation. Thromb. Haemost. 94, 341–346 (2005). 16113824 10.1160/TH05-01-0053

[b43] Fatokun A. A., Hunt N. H. & Ball H. J. Indoleamine 2,3-dioxygenase 2 (IDO2) and the kynurenine pathway: characteristics and potential roles in health and disease. Amino Acids 45, 1319–1329 (2013). 24105077 10.1007/s00726-013-1602-1

[b44] Brandacher G. *et al.* Bariatric surgery cannot prevent tryptophan depletion due to chronic immune activation in morbidly obese patients. Obes Surg 16, 541–548 (2006). 16687019 10.1381/096089206776945066

[b45] Moriya A., Fukuwatari T., Sano M. & Shibata K. Different variations of tissue B-group vitamin concentrations in short- and long-term starved rats. Br. J. Nutr. 107, 52–60 (2012). 21733331 10.1017/S0007114511002339

[b46] Grases F., Garcia-Ferragut L. & Costa-Bauza A. Development of calcium oxalate crystals on urothelium: effect of free radicals. Nephron 78, 296–301 (1998). 9546690 10.1159/000044939

[b47] Urivetzky M., Kessaris D. & Smith A. D. Ascorbic acid overdosing: a risk factor for calcium oxalate nephrolithiasis. J. Urol. 147, 1215–1218 (1992). 1569652 10.1016/s0022-5347(17)37521-3

[b48] Kumar C. K., Yanagawa N., Ortiz A. & Said H. M. Mechanism and regulation of riboflavin uptake by human renal proximal tubule epithelial cell line HK-2. Am. J. Physiol. 274, F104–F110 (1998). 9458829 10.1152/ajprenal.1998.274.1.F104

[b49] Said H. M., Ortiz A. & Vaziri N. D. Mechanism and regulation of vitamin B(6) uptake by renal tubular epithelia: studies with cultured OK cells. Am. J. Physiol. Renal Physiol. 282, F465–F471 (2002). 11832427 10.1152/ajprenal.00267.2001

[b50] Verri A., Laforenza U., Gastaldi G., Tosco M. & Rindi G. Molecular characteristics of small intestinal and renal brush border thiamin transporters in rats. Biochim. Biophys. Acta 1558, 187–197 (2002). 11779568 10.1016/s0005-2736(01)00430-8

[b51] Huo W., Zhang K., Nie Z., Li Q. & Jin F. Kidney injury molecule-1 (KIM-1): a novel kidney-specific injury molecule playing potential double-edged functions in kidney injury. Transplant Rev (Orlando) 24, 143–146 (2010). 20447817 10.1016/j.trre.2010.02.002

[b52] Smith C. A., Want E. J., O’Maille G., Abagyan R. & Siuzdak G. XCMS: processing mass spectrometry data for metabolite profiling using nonlinear peak alignment, matching, and identification. Anal. Chem. 78, 779–787 (2006). 16448051 10.1021/ac051437y

[b53] Smilde A. K., van der Werf M. J., Bijlsma S., van der Werff-van der Vat B. J. & Jellema R. H. Fusion of mass spectrometry-based metabolomics data. Anal. Chem. 77, 6729–6736 (2005). 16223263 10.1021/ac051080y

[b54] Chan E. C., Pasikanti K. K. & Nicholson J. K. Global urinary metabolic profiling procedures using gas chromatography-mass spectrometry. Nat Protoc 6, 1483–1499 (2011). 21959233 10.1038/nprot.2011.375

