# Supplementary material for: Metabolomics analysis for hydroxy-L-proline-induced calcium oxalate nephrolithiasis in rats based on ultra-high performance liquid chromatography quadrupole time-of-flight mass spectrometry
Source: Sci Rep. 2016 Jul 22;6:30142. doi: 10.1038/srep30142 (PMC4957101; doi:10.1038/srep30142)
Supplement: Supplementary Information [file srep30142-s1.doc]

**Metabolomics analysis for hydroxy-L-proline-induced calcium oxalate nephrolithiasis in rats based on ultra-high performance liquid chromatography quadrupole time-of-flight mass spectrometry**

Songyan Gao^1, §^, Rui Yang^4^, ^§^, Zhongjiang Peng^2^, Hongtao Lu^4^, Na Li^1^, Jiarong Ding^2^, Xingang Cui^3,*^, Wei Chen^2,*^ and Xin Dong^1,*^

^1^ School of Pharmacy, Second Military Medical University, Shanghai 200433, China

^2^ Department of Nephrology, Changhai Hospital, Shanghai 200433, China

^3^ Department of Urology, The Third Affiliated hospital, Second Military Medical University, Shanghai 200433, China

^4^ Brigade of undergraduate student, Second Military Medical University, Shanghai 200433, China

^§^ These authors contributed equally to this work.

^*^ Correspondence Author**,** Xin Dong: [dongxinsmmu@126.com](mailto:dongxinsmmu@126.com)**,** Wei Chen: [chenwei@smmu.edu.cn](mailto:chenwei@smmu.edu.cn) **,** Xingang Cui: cuixingang@163.com


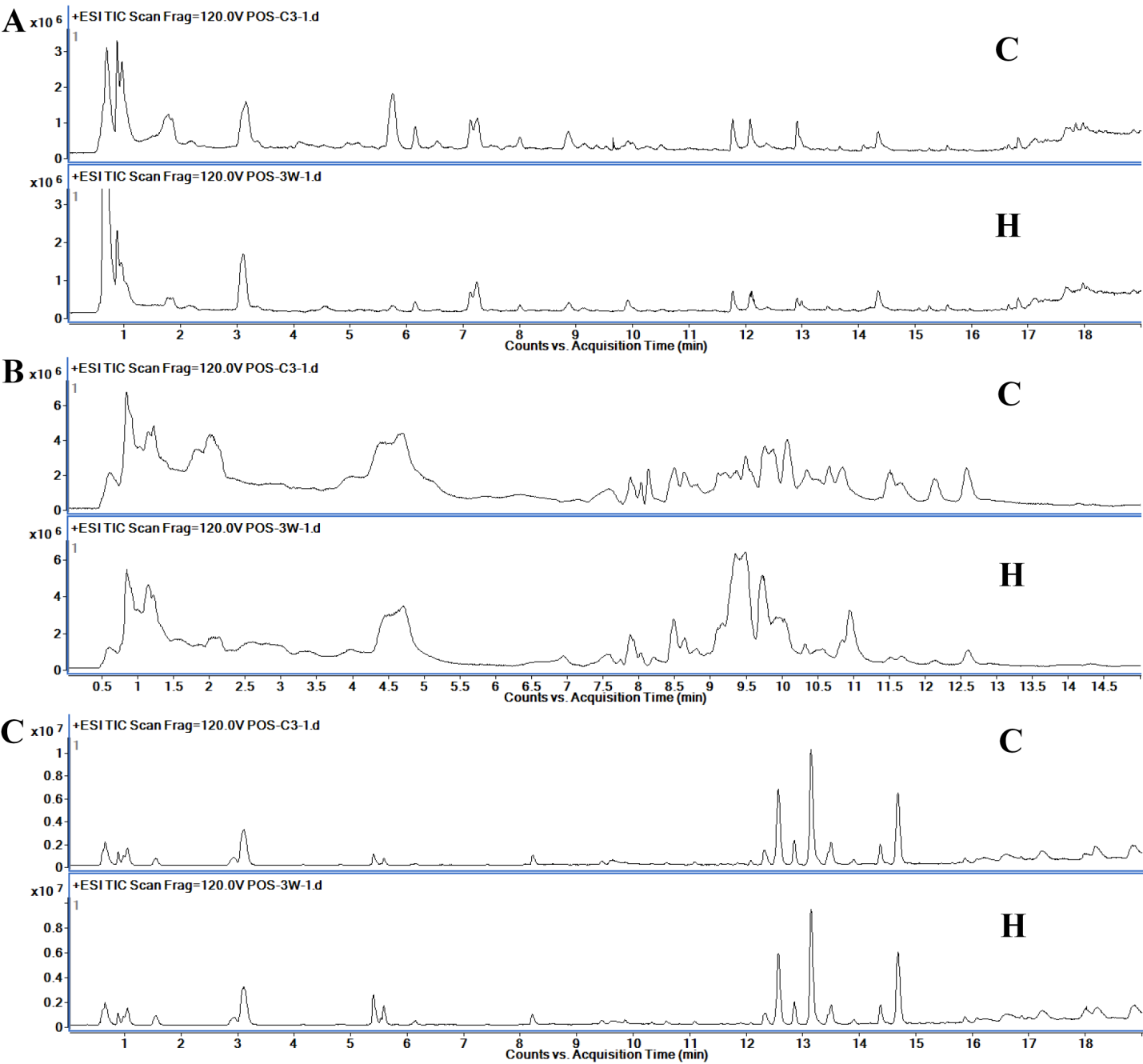


**Figure S1.** Typical total ion chromatograms (TICs) of the urine samples based on the RPLC-MS (A) and HILIC-MS methods (B) and the serum samples based on the RPLC-MS methods (C) in ESI positive mode of C and H groups.


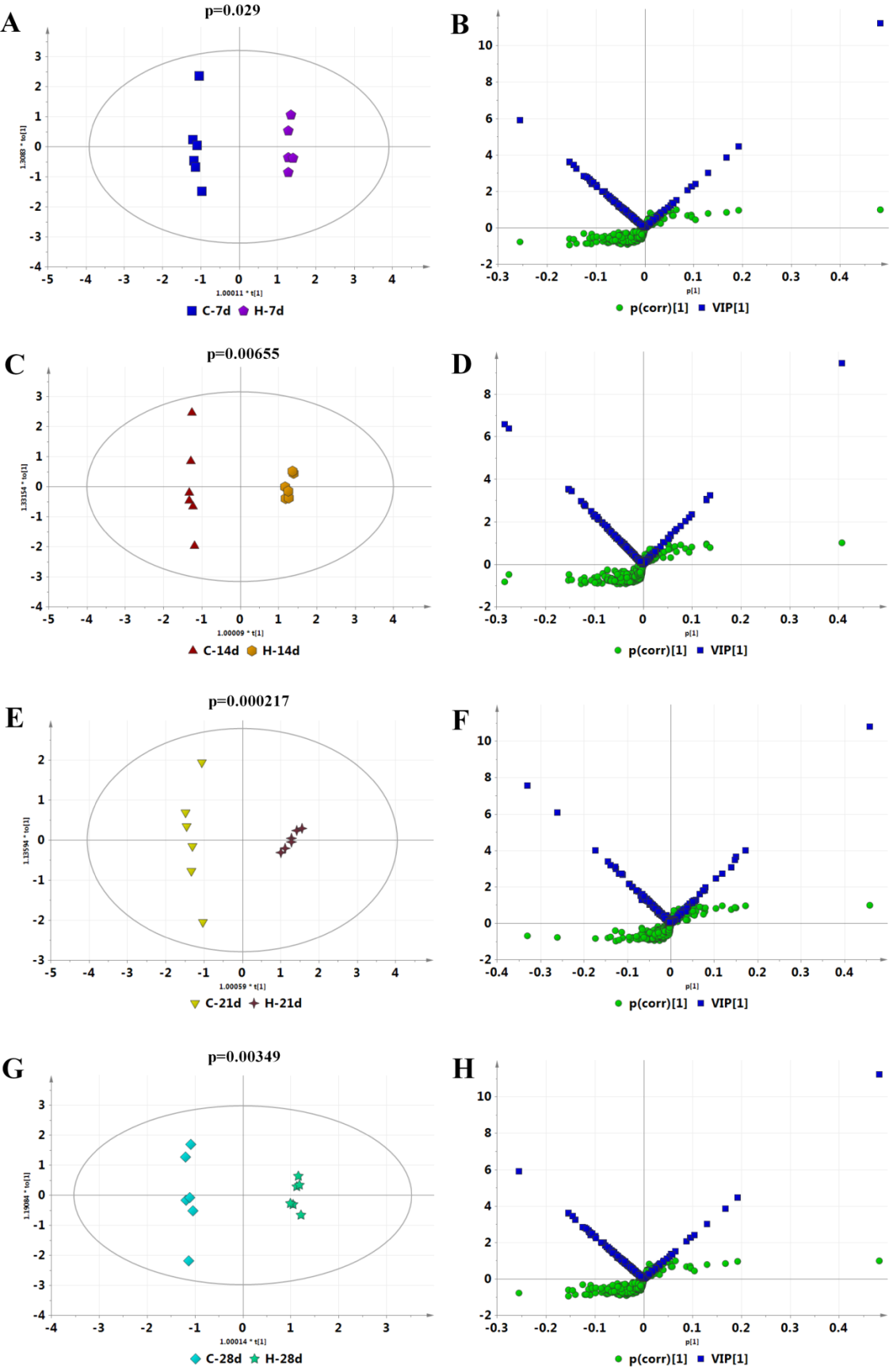


**Figure S2.** OPLS-DA score plot and s-plot of the C and H rats at each time point by RPLC-MS method.


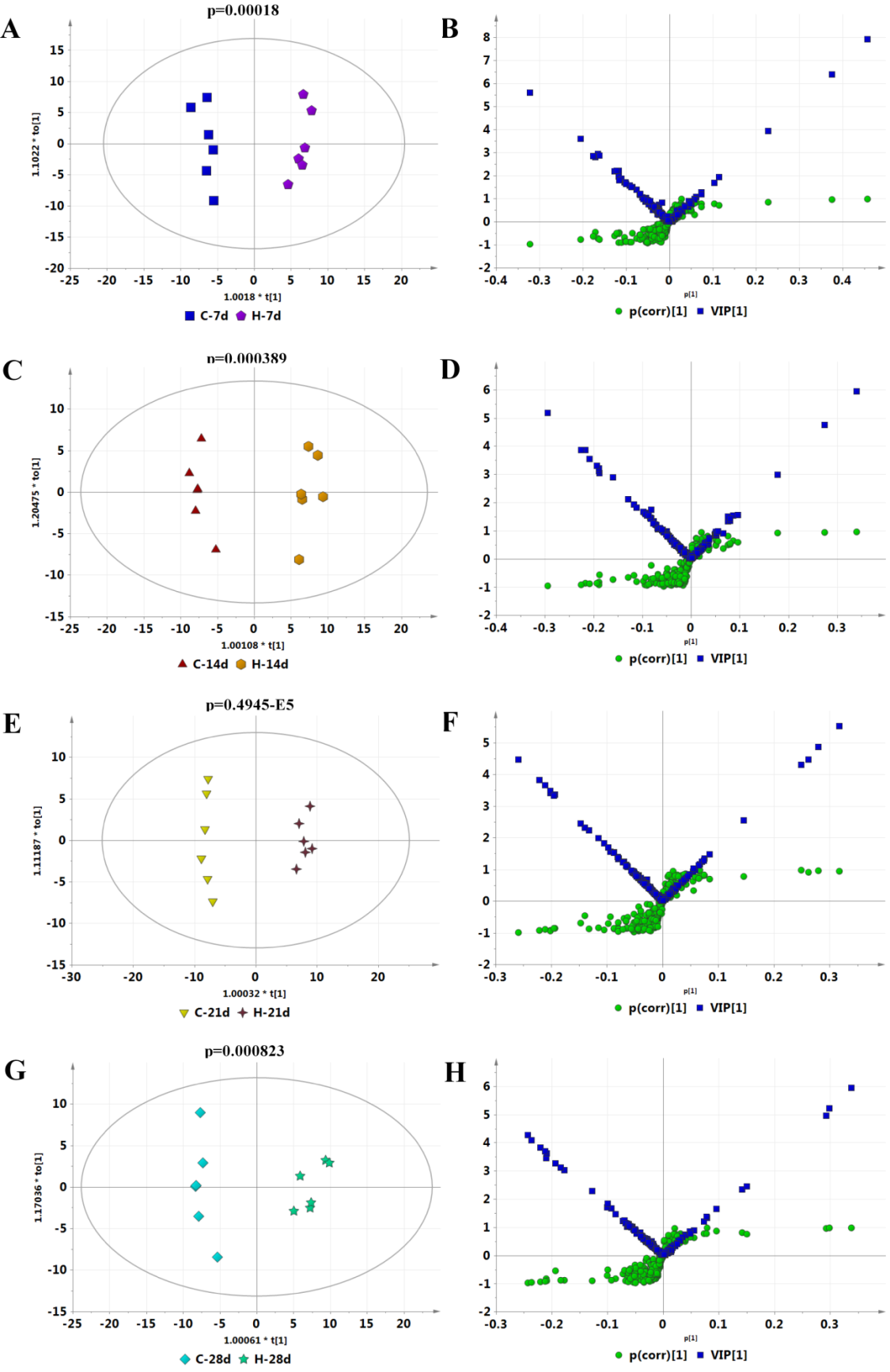


**Figure S3.** OPLS-DA score plot and s-plot of the C and H rats at each time point by HILIC-MS method.


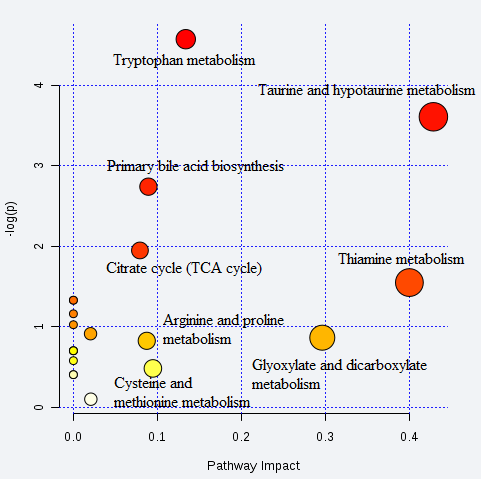


**Figure S4.** The summary of pathway analysis based on IPA analysis using the MetaboAnalyst platform.

**Supplementary Table 1.** Results of ESI model U-HPLC-Q-TOF/MS for the discriminative urinary metabolites of HLP-induced HLP-induced crystal renal injury

| **No.** | **Mode** | **m/z** | **RT/min** | **Metabolite** | **Foumula** | **Ion** | **Main Fragments** |
| --- | --- | --- | --- | --- | --- | --- | --- |
| 1 | HILIC | 116.0707 | 9.15 | L-Proline | C5H9NO2 | [M+H]+ | 68.0492,70.0657,71.0691,99.0632 |
| 2 | RP | 118.0720 | 0.71 | Betaine | C5H11NO2 | [M+H]+ | 76.0399,71.0691,59.0730,58.0654 |
|  | HILIC | 118.0861 | 9.74 | Betaine | C5H11NO2 | [M+H]+ | 58.0653,59.073 |
| 3 | RP | 123.0440 | 8.87 | Benzoic acid | C7H6O2 | [M+H]+ | 105.0336,80.0578，79.0545,77.0389，51.0234 |
| 4 | RP | 124.0080 | 0.66 | Taurine | C2H7NO3S | [M-H]- | 106.9795，94.9804，79.9575 |
| 5 | RP | 124.0370 | 0.89 | Nicotinic acid | 6H5NO2 | [M+H]+ | 106.0284,98.0601,80.0499,78.0343,53.0389 |
| 6 | RP | 127.0390 | 2.44 | Maltol | C6H6O3 | [M+H]+ | 109.0280,81.0340,55.0182,53.0386 |
| 7 | HILIC | 130.0865 | 9.20 | L-Pipecolic acid | C6H11NO2 | [M+H]+ | 56.0502,68.0501,70.0657,84.0812 |
| 8 | HILIC | 131.1180 | 9.08 | N-Acetylputrescine | C6H14N2O | [M+H]+ | 55.0549,60.0451,72.0810,86.0604,114.0916,115.0951 |
| 9 | HILIC | 134.0582 | 0.60 | Indoxyl | C8H7NO | [M+H]+ | 79.053,106.0348,116.0467 |
|  | RP | 134.0600 | 4.22 | Indoxyl | C8H7NO | [M+H]+ | 106.065,79.0542 |
| 10 | RP | 137.0600 | 9.12 | Phenylacetic acid | C8H8O2 | [M+H]+ | 91.0545,65.0390 |
| 11 | HILIC | 138.0547 | 9.88 | Trigonelline | C7H7NO2 | [M+H]+ | 94.0659, 92.0497, 78.0344 |
| 12 | RP | 144.0310 | 0.70 | 2/4-Oxoglutaramate | C5H7NO4 | [M-H]- | 129.0189,116.0349,101.0245,85.0297,72.0453,57.0349 |
| 13 | RP | 144.0850 | 0.71 | Proline betaine | C7H13NO2 | [M+H]+ | 126.0911,97.0761,84.0813,60.0559,58.0660 |
| 14 | HILIC | 146.1176 | 9.10 | 3-Dehydroxycarnitine | C7H15NO2 | [M+H]+ | 58.0649,60.0809,87.0443, 100.1112 |
| 15 | RP | 150.0540 | 0.88 | L-Methionine | C5H11NO2S | [M+H]+ | 133.0316,104.0530,86.0603,74.0604,61.0108,56.0498 |
| 16 | RP | 154.0500 | 1.55 | 5-Aminosalicylic Acid | C7H7NO3 | [M+H]+ | 136.0391，108.0439 |
| 17 | RP | 162.0550 | 6.16 | Indole-3-carboxylic acid | C9H7NO2 | [M+H]+ | 144.0437, 116.0494, 89.0384 |
| 18 | RP | 162.0550 | 8.01 | 2-Indolecarboxylic acid | C9H7NO2 | [M+H]+ | 144.0439, 120.0430, 116.0493, 101.0391, 89.0388 |
| 19 | RP | 162.0560 | 7.16 | 3-Methyldioxyindole | C9H9NO2 | [M-H]- | 144.0448, 134.0608, 132.0457, 120.0451, 108.0455, 91.0185 |
|  | RP | 164.0700 | 7.13 | 3-Methyldioxyindole | C9H9NO2 | [M+H]+ | 146.0615,122.0595,91.0538,55.0179 |
| 20 | HILIC | 163.0257 | 1.70 | Succinic acid | C4H6O4 | [M+FA-H]- | 55.0193，72.9939,99.0081,117.0176,145.0139 |
|  | RP | 163.0260 | 0.69 | Succinic acid | C4H6O4 | [M+FA-H]- | 145.0146,117.0203,99.0091,75.0100,57.0356 |
| 21 | RP | 166.0690 | 0.89 | 7-Methylguanine | C6H7N5O | [M+H]+ | 150.0543,149.0456,124.0495,109.0791 |
| 22 | RP | 167.0220 | 0.89 | Uric acid | C5H4N4O3 | [M-H]- | 124.0156,96.0204,78.0353,69.0094 |
| 23 | RP | 167.1070 | 7.28 | Decatrienoic acid | C10H14O2 | [M+H]+ | 149.0972，121.1003,107.0854,79.0543,67.0552,57.0328 |
| 24 | RP | 172.9930 | 3.36 | Phenol sulfate | C6H6O4S | [M-H]- | 109.0272,93.0351,79.9577 |
| 25 | RP | 186.0560 | 11.84 | Indoleacrylic acid | C11H9NO2 | [M-H]- | 142.0663, 116.0507 |
|  | RP | 188.0700 | 11.84 | Indoleacrylic acid | C11H9NO2 | [M+H]+ | 170.0589,142.0644,115.0543 |
| 26 | RP | 187.0080 | 6.75 | p-Cresol sulfate | C7H8O4S | [M-H]- | 169.0886, 141.8632, 107.0501, 79.9576 |
| 27 | RP | 188.0350 | 3.88 | Kynurenic acid | C10H7NO3 | [M-H]- | 144.0455,109.0297,79.9584 |
|  | RP | 190.0500 | 3.83 | Kynurenic acid | C10H7NO3 | [M+H]+ | 172.0394,144.0444，116.0491,89.0387 |
| 28 | HILIC | 188.9845 | 0.57 | Pyrocatechol sulfate | C6H6O5S | [M-H]- | 53.0408,79.9584,109.0302 |
|  | RP | 188.9870 | 2.66 | Pyrocatechol sulfate | C6H6O5S | [M-H]- | 109.0296,79.9580 |
| 29 | RP | 190.0540 | 3.42 | N-Acetyl-DL-methionine | C7H13NO3S | [M-H]- | 148.0430,142.0515,98.0609 |
|  | RP | 192.0690 | 3.37 | N-Acetyl-DL-methionine | C7H13NO3S | [M+H]+ | 150.058,144.0657,133.0311,104.0530,98.0597 |
| 30 | RP | 191.0210 | 0.71 | Citric acid/Isocitric acid | C6H8O7 | [M-H]- | 175.0195,129.0189,111.0089,87.0097,57.0361 |
| 31 | HILIC | 192.0674 | 1.12 | Phenylacetylglycine | C10H11NO3 | [M-H]- | 74.0255 |
|  | RP | 192.0680 | 5.78 | Phenylacetylglycine | C10H11NO3 | [M-H]- | 74.0255 |
|  | RP | 194.0830 | 5.76 | Phenylacetylglycine | C10H11NO3 | [M+H]+ | 176.0707,148.0756,120.0807,91.0546,76.0397 |
|  | RP | 216.0630 | 5.75 | Phenylacetylglycine | C10H11NO3 | [M+Na]+ | —— |
| 32 | RP | 201.1130 | 12.16 | Sebacic acid | C10H18O4 | [M-H]- | 183.1012，164.8359, 139.1131, 111.0815, 57.0337 |
| 33 | HILIC | 203.1504 | 14.32 | ADMA | C8H18N4O2 | [M+H]+ | 88.0866,116.0710, 158.1290 |
| 34 | RP | 204.0300 | 3.03 | Xanthurenic acid | C10H7NO4 | [M-H]- | 174.1726,160.0412,124.0408,116.0493,79.9557 |
|  | RP | 206.0450 | 2.94 | Xanthurenic acid | C10H7NO4 | [M+H]+ | 188.0342,160.0393,132.0448 |
| 35 | RP | 208.0620 | 2.16 | Hydroxyphenylacetylglycine | C10H11NO4 | [M-H]- | 133.0296,74.025 |
| 36 | RP | 211.1080 | 1.37 | L-prolyl-L-proline | C10H16N2O3 | [M-H]- | 114.0549, 98.0609, 86.0593, 70.0655 |
| 37 | RP | 212.0040 | 4.31 | Indoxylsulfuric acid | C8H7NO4S | [M-H]- | 132.0454,104.0506,79.9580 |
| 38 | RP | 212.0900 | 0.71 | 3-Methoxytyrosine | C10H13NO4 | [M+H]+ | 194.0937, 170.0929, 166.096, 124.0866, 109.0753, 70.0643 |
| 39 | RP | 220.1190 | 1.85 | Pantothenic Acid | C9H17NO5 | [M+H]+ | 202.1069,184.0970,142.0856,124.0753,90.0553,72.0446 |
|  | RP | 242.1000 | 1.85 | Pantothenic Acid | C9H17NO6 | [M+Na]+ | —— |
| 40 | RP | 254.9830 | 0.73 | Ascorbate 2-sulfate | C6H8O9S | [M-H]- | 175.0249，115.0036,71.0143 |
| 41 | HILIC | 265.1124 | 11.68 | Thiamine | C12H16N4OS | [M+H]+ | 81.0443,122.0709,144.0473 |
| 42 | RP | 377.1450 | 6.54 | Riboflavin | C17H20N4O6 | [M+H]+ | 243.0879,172.0859,99.0438,69.0331,57.0336 |

**Supplementary Table 2.** Results of ESI model U-HPLC-Q-TOF/MS for the discriminative serum metabolites of HLP-induced HLP-induced crystal renal injury

| **No.** | **m/z** | **RT/min** | **Metabolite** | **Formula** | **Ion** | **Main Fragments** |
| --- | --- | --- | --- | --- | --- | --- |
| 1 | 117.0193 | 0.98 | Succinic acid | C4H6O4 | [M-H]- | 99.0083,73.0299,55.0191 |
| 2 | 130.0119 | 0.59 | 4-Hydroxy-L-proline | C5H9NO3 | [M-H]- | —— |
| 3 | 158.0818 | 3.35 | Valerylglycine | C7H13NO3 | [M-H]- | —— |
| 4 | 162.1127 | 0.68 | L-Carnitine | C7H15NO3 | [M+H]+ | 103.0387,102.0916,85.0285,60.0807,57.0334 |
|  | 184.0950 | 0.64 | L-Carnitine | C7H15NO3 | [M+Na]+ | 125.0201 |
| 5 | 204.0661 | 5.78 | Indolelactic acid | C11H11NO3 | [M-H]- | —— |
| 6 | 209.0922 | 1.49 | Kynurenine | C10H12N2O3 | [M+H]+ | 192.0659,174.056,163.0861,150.0549,146.0592,136.0748,120.0438,94.0651,74.0227 |
| 7 | 218.1031 | 1.85 | Pantothenic Acid | C9H17NO5 | [M-H]- | 146.0825,88.0402,71.0139 |
|  | 220.1181 | 1.85 | Pantothenic Acid | C9H17NO5 | [M+H]+ | 202.1077,184.0955,174.1092,142.0846,124.0761,116.0339,90.0545,72.0440 |
| 8 | 218.1389 | 1.08 | Propionylcarnitine | C10H19NO4 | [M+H]+ | 159.0638,144.0988,99.0129,85.0282,60.0805 |
| 9 | 246.1702 | 4.19 | 2-Methylbutyroylcarnitine | C12H23NO4 | [M+H]+ | 187.0956,144.1010,85.0285,60.0808 |
| 10 | 253.2168 | 15.75 | Hexadecenoic Acid | C16H30O2 | [M-H]- | —— |
|  | 255.2303 | 15.74 | Hexadecenoic Acid | C16H30O2 | [M+H]+ | 237.2219,219.2085,163.1474,149.1302,135.1177 |
| 11 | 355.2635 | 9.86 | Cholic acid | C24H40O5 | Fragment | 337.2505,299.1992,245.1550,213.1638,159.1163,109.1008 |
|  | 373.2742 | 9.86 | Cholic acid | C24H40O5 | Fragment | 355.2639,337.2542,319.1949,273.1853,245.1548 |
|  | 407.2806 | 9.87 | Cholic acid | C24H40O5 | [M-H]- | 343.2643,325.2535,289.2178,251.1012,207.1771123.0818,95.0505,69.0348 |
|  | 426.3215 | 9.86 | Cholic acid | C24H40O5 | [M+NH4]+ | —— |
|  | 443.2569 | 9.87 | Cholic acid | C24H40O5 | [M+Cl]- | 407.2797 |
| 12 | 498.2894 | 8.12 | Tauroursodeoxycholic acid | C26H45NO6S | [M-H]- | 124.0070,106.9819,79.9580 |
| 13 | 514.2842 | 7.31 | Taurocholic acid | C26H45NO7S | [M-H]- | —— |
